# SCAGC-UNet: Graph Convolutional Network with Spatial and Channel Attention for Medical Image Segmentation

**DOI:** 10.3390/jimaging12070302

**Published:** 2026-07-06

**Authors:** Xiaolong Hu, Xueyan Liu, Junji Jiang, Ziqi Hao, Lishan Qiao

**Affiliations:** 1 School of Mathematical and Systems Science, Liaocheng University, Liaocheng 252000, China; hxl6373@163.com (X.H.); jiangjunji017@163.com (J.J.); lcuhzq@163.com (Z.H.); 2 School of Computer Science and Technology, Shandong Jianzhu University, Jinan 250101, China; 24455@sdjzu.edu.cn

**Keywords:** medical image segmentation, graph convolutional networks, spatial-channel attention

## Abstract

Medical image segmentation is critical for clinical diagnosis, yet existing methods face a persistent trade-off: CNN-based approaches are constrained by local receptive fields, while Transformer-based methods suffer from semantic dilution when modeling global context. To address these limitations, we propose SCAGC-UNet, a region-aware graph convolutional network that bridges local detail extraction and global dependency modeling through structured region-level reasoning. The architecture features a dual-layer residual encoder for hierarchical feature extraction and a Spatial-Channel Graph Convolution (SC-GCN) module at the bottleneck, which simultaneously captures inter-region spatial topology and intra-region channel semantics via dual-branch graph inference. Feature refinement in the decoder is further enhanced by Context-Corrected Modules and Backward-Aided Modules to reduce the semantic gap across skip connections. We validate SCAGC-UNet on three public benchmarks covering distinct imaging challenges. On Kvasir-SEG, the model achieves a Dice score of 92.28% and MIOU of 92.41%, surpassing the strongest CNN-based baseline CCBANet by 0.73% in DSC and outperforming TransUNet by 11.76% in DSC. On BUSI, it attains an IOU of 78.10% and MIOU of 87.68%, outperforming UNet by 2.82% in IOU and TransUNet by 6.91% in DSC. On COVID-19 CT, it achieves a DSC of 82.51%, surpassing UNet by 4.99% and TransUNet by 7.47%, demonstrating robust performance on irregular lesion morphologies. These results confirm that SCAGC-UNet achieves consistent and robust segmentation performance across three public benchmark datasets spanning distinct imaging modalities, suggesting its potential clinical relevance.

## 1. Introduction

Medical image segmentation is a fundamental task in intelligent healthcare systems, providing essential structural and pathological information for diagnosis, treatment planning, and prognosis assessment. With the rapid advancement of medical imaging technologies and computational resources, automated segmentation methods have become indispensable in clinical workflows. However, medical images are characterized by high anatomical variability, low contrast, blurred boundaries, and small lesion regions, making accurate segmentation particularly challenging. These properties require models capable of simultaneously capturing fine-grained local details and long-range contextual dependencies.

In recent years, convolutional neural networks (CNNs), particularly UNet [1], have achieved remarkable success in medical image segmentation due to their local perception capabilities and hierarchical feature extraction. Although CNN-based variants address certain limitations from different perspectives, their fundamental operations remain confined to local pixel or feature map processing [2], making it difficult to overcome the structural limitation of local perception. To overcome this issue, the Vision Transformer [3] has introduced a new paradigm for computer vision by modeling global dependencies via self-attention mechanisms. On this basis, Transformer-based architectures have emerged as a major research direction for visual tasks [4], effectively addressing the inherent drawback of CNNs in capturing long-range dependencies.

However, the global attention mechanism in Transformers introduces computational redundancy and semantic dilution that are particularly problematic in the medical imaging context. Through representational similarity analysis using Centered Kernel Alignment, Raghu et al. [5] demonstrate that ViT aggregates global information even at its earliest layers, producing representations that remain highly uniform across all network depths. This stands in sharp contrast to CNNs, whose receptive fields expand gradually from local to global scales. Critically, neither architecture exhibits a structured intermediate stage in which representations are organized around structured, feature-coherent spatial regions: CNNs are bound to local neighborhoods in early layers, while ViTs immediately collapse to an image-level global context. As a consequence, when images are flattened into token sequences in Transformer-based medical image segmentation models, local boundary structures and regional coherence are weakened. Shamshad et al. [6] further note in their comprehensive survey of Transformers in medical imaging that global self-attention’s lack of spatial inductive bias poses persistent challenges for localizing small and low-contrast lesions, whose discriminative features can be subsumed by background noise under uniform global aggregation. Furthermore, standard self-attention operates primarily in the spatial dimension, lacking explicit mechanisms to model inter-channel relationships. As a result, small, low-contrast lesion regions with weak boundaries tend to be misclassified as background, a failure mode that is particularly pronounced when channel-level semantic consistency is not jointly considered.

Both CNN local convolutions and Transformer global self-attention overlook an intermediate semantic level: the region. Unlike a simple local patch (a fixed-size crop without semantic awareness) or a superpixel (defined purely by low-level color and intensity similarity), a region in our formulation refers to an encoder-derived spatial partition of the bottleneck feature map, which achieves a practical approximation to semantic coherence through the hierarchical pooling of the deep encoder. Rather than being defined by explicit semantic boundaries, each partition groups feature vectors whose deep representations exhibit high intra-region cosine similarity—a consequence of the encoder’s large receptive field, which causes semantically related content to cluster in adjacent feature map locations. Such regions retain local detail precision while offering global context interpretability, serving as a semantic bridge between pixel-level locality and image-level global context. The absence of explicit regional modeling in existing architectures means that neither CNN nor Transformer can fully capture structured dependencies among semantically meaningful spatial units—a gap that becomes especially critical when distinguishing lesions from visually similar surrounding tissues.

Graph Neural Networks (GNNs) provide a natural and principled framework for modeling such region-level structures. A graph representation allows encoder-derived spatial partitions to be treated as nodes, with edges encoding their topological adjacency and feature-space affinity. This formulation explicitly preserves the relational structure among regions—something that neither sliding-window convolutions nor flattened token sequences can achieve. Moreover, GNNs can deeply integrate both graph topology and node attributes [7,8], enabling knowledge propagation and reasoning across local and global scales [9,10,11]. By leveraging graph structure and node connections, GNNs capture comprehensive contextual features and distant dependencies while maintaining fewer parameters than Transformer modules. However, existing GNN-based segmentation models apply graph modules without explicitly defining structured, feature-coherent spatial partitions as graph nodes, failing to fully leverage the complementary connections between spatial and channel features.

We propose a hybrid network architecture based on region-aware graph reasoning. It achieves collaborative modeling of local details, regional semantics, and global context through an enhanced residual encoder and a dual-branch graph reasoning module. The encoder uses a dual-layer residual convolutional structure that ensures stable gradient propagation while enhancing local feature extraction. This mitigates detail decay in deep networks, preserving sufficient low-level semantics for regional modeling. To address insufficient regional feature modeling, we introduce a block-based graph inference mechanism. Encoder-derived spatial partitions with high intra-region feature consistency are defined as graph nodes, constructing a space-channel dual-branch graph convolutional network. The spatial branch characterizes topological relationships between regions to model structural dependencies between lesions and surrounding tissues. The channel branch extracts semantic consistency across channels within regions. This dual approach enhances the network’s ability to distinguish low-contrast lesions with weak boundaries. We also propose a residual dynamic graph convolutional feature extraction module based on DeepGCN [12] principles.

Our key contributions are:

(1) We propose a region-aware graph inference framework that explicitly models encoder-derived spatial partitions as feature-coherent graph nodes, bridging pixel-level locality and image-level global context.
(2) We introduce a dual-branch graph architecture that synergistically captures spatial topological relationships between regions and channel semantic consistency within regions.
(3) We develop deep graph convolutional modules that effectively propagate region-level features while addressing vanishing gradients in DeepGNN architectures.
(4) Extensive experiments across three imaging modalities (endoscopy, CT, ultrasound) demonstrate state-of-the-art performance and strong generalization.

The remainder of this paper is organized as follows: Section 2 reviews related work. Section 3 describes the proposed a Spatial-Channel Attention Graph Convolution UNet (SCAGC-UNet) architecture in detail. Section 4 presents experimental settings and quantitative results. Section 5 provides ablation studies and qualitative analysis. Section 6 concludes the paper and discusses future directions.

## 2. Related Work

### 2.1. CNN-Based Medical Image Segmentation

Medical image segmentation has evolved significantly with the development of deep learning techniques. Early fully convolutional architectures [13] laid the foundation for end-to-end pixel-wise prediction. Among them, UNet [1] introduced an encoder–decoder framework with skip connections, effectively leveraging feature information at different levels while preserving shallow features. Subsequent works have improved upon UNet along several dimensions. In terms of skip connection design, Zhou et al. argued that UNet’s skip connections were too simple and proposed UNet++ with nested and dense skip connections [14], which better captures features at different scales and preserves details of foreground objects. Fateme Hoorali et al. further observed that original skip connections only aggregate features of the same scale and proposed TRUNet [15], which integrates multi-scale features and residual networks into skip connections to improve segmentation quality.

In terms of encoder design, Sahadev Poudel et al. noted that UNet has limited ability to capture global features and adopted EfficientNetB0 [16] as the encoder, developing an attention module to suppress noise and irrelevant regions [17]. To address low resolution in 2D slice images and challenges in small tumor segmentation, Guotai Wang et al. proposed 2.5D UNet [18], which captures correlations and contextual information between feature channels under low computational constraints. Despite these advances, CNN-based methods remain fundamentally constrained by local receptive fields, limiting their capacity to model long-range dependencies and region-level contextual relationships.

### 2.2. Transformer-Based Medical Image Segmentation

Building on Vision Transformer principles [3], Transformer-based architectures have substantially advanced medical image segmentation. Chen et al. proposed TransUNet [19], which combines CNN feature extraction with Transformer-based global attention, establishing a widely adopted hybrid paradigm. Liu et al. proposed the Swin Transformer with shifted windows [20], which incorporates hierarchical attention mechanisms and the inductive bias of CNNs while leveraging self-attention to capture long-range dependencies. Kai Han et al. introduced Transformer-in-Transformer (TNT) [21] to model both patch-level and pixel-level features, addressing the neglect of intra-patch structural information in standard Transformers. To better combine multi-scale CNN and Transformer features, Yuan et al. proposed a CNN–Transformer complementary network [22] that jointly exploits local convolutional representations and global self-attention for improved segmentation. Yinghua Fu et al. further proposed TSCA-Net [23], which introduces spatial-channel attention to enhance feature discrimination in hybrid architectures.

Despite their success in global context modeling, Transformer-based methods share two critical limitations in the medical imaging context: flattening images into token sequences weakens local boundary structures and regional coherence, leading to semantic dilution for small lesions, and standard self-attention operates in the spatial dimension without explicit inter-channel relationship modeling, reducing sensitivity to channel-level semantic consistency that is important for distinguishing low-contrast lesions. These limitations motivate the need for an intermediate regional modeling layer that neither paradigm currently provides.

### 2.3. GNN-Based Region-Level Reasoning

Graph convolutional approaches have been applied to semantic segmentation to model non-local relationships between regions, enabling structured reasoning beyond local receptive fields [24,25]. To support deeper and more expressive graph networks, Li et al. proposed DeepGCN [12], which adapts residual connections and dilated convolutions from CNNs to GNNs, effectively alleviating over-smoothing and gradient vanishing in stacked graph convolutional layers.

In the context of medical image segmentation, several works have explored GNN-based approaches to enhance feature interaction. Juntong Fan et al. proposed DSFNet [26], which integrates a Dual-GCN module at the bottleneck layer using shortest-path graph networks to enhance global context fusion. Md Mostafijur Rahman et al. introduced the Cascade Graph Convolution Attention Decoder [27], which refines long-range dependencies using global receptive fields of graph convolutional blocks. Both methods focus on global contextual aggregation but treat the entire feature map as a monolithic graph input, without partitioning it into structured, feature-coherent spatial partitions. Qihang Ma et al. proposed DGRUnit [28], which jointly models spatial topology and channel dependencies via GCN and GAT components. While DGRUnit advances toward dual-dimensional reasoning, its graph is constructed directly on pixel-level feature maps: nodes correspond to individual spatial positions rather than pooled encoder-derived spatial regions, which limits the model’s ability to capture structured inter-region relationships and intra-region channel consistency simultaneously. Moreover, pixel-level graph construction incurs a computational cost of $O({\left(HW\right)}^{2})$ with respect to spatial resolution: for a 192 × 192 input, this amounts to approximately 1.35 × 10^9^ pairwise entries if applied to the full-resolution feature map, and approximately 20,736 entries at the bottleneck level (12 × 12 spatial resolution after four downsampling stages)—scaling quadratically with input resolution and posing fundamental scalability constraints. In contrast, the SC-GCN module constructs the graph over S=9 region nodes (with patch size p=4 on a 12 × 12 bottleneck), yielding an adjacency matrix of only $S^{2}=81$ entries with complexity $O(S^{2})$—a 99.6% reduction compared to the bottleneck-level pixel graph. This compact graph design enables global relational reasoning across the full spatial extent of the feature map while remaining computationally scalable [29].

The above works share a common limitation: graph modules are applied to raw or pooled feature maps without defining structured, feature-coherent spatial partitions as the basic graph units. As a result, neither spatial topological relationships between regions nor intra-regional channel semantic consistency are jointly and explicitly modeled. Our work addresses this gap by constructing graphs over encoder-derived spatial partitions that exhibit high intra-region feature consistency, and further introducing a dual-branch architecture that simultaneously captures inter-region spatial topology and intra-region channel semantics, as detailed in Section 3.

## 3. Methods

**Figure 1:**
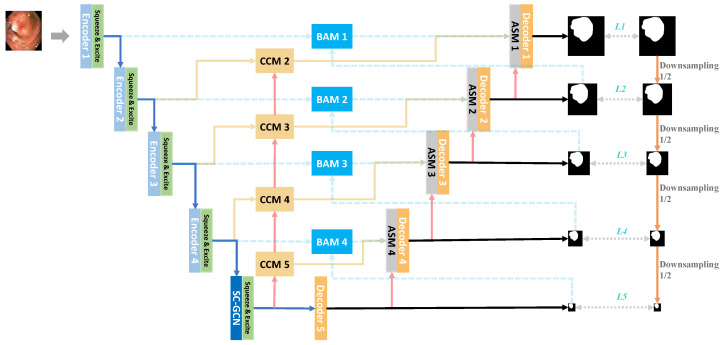
Overall architecture of SCAGC-UNet. The encoder progressively extracts hierarchical features through dual-layer residual modules (Layers 1–4) and a region-aware graph inference module at the bottleneck (Layer 5). The decoder reconstructs segmentation masks through multi-scale feature fusion with skip connections enhanced by CCM and BAM modules.

We propose SCAGC-UNet, whose overall architecture is illustrated in Figure 1. The network adopts a five-layer encoder–decoder structure. In the encoder, the first four layers employ dual-layer residual modules combined with Squeeze-and-Excitation (SE) blocks to extract multi-scale local features while adaptively recalibrating channel-wise feature responses. At the bottleneck layer, we replace the residual modules with the proposed Spatial-Channel Graph Convolution (SC-GCN) module, which is better suited for modeling region-level semantic relationships given its largest receptive field among all layers. In the decoder, to mitigate semantic discrepancies between encoder and decoder features, we incorporate Context Comparison Modules (CCMs) and Bottleneck Attention Modules (BAMs) at each layer [30], where CCM acquires contextual information from encoder features and BAM leverages decoder predictions to refine feature extraction through backward guidance. In addition, Auxiliary Scale Modules (ASMs) [31] are introduced to selectively fuse multi-scale features across encoder layers, enabling the decoder to effectively integrate information at different scales. Finally, deep supervision is applied by generating predictions at each decoder layer, with the final output taken from the highest decoder layer.

### 3.1. Dual-Layer Residual Encoder Module

**Figure 2:**
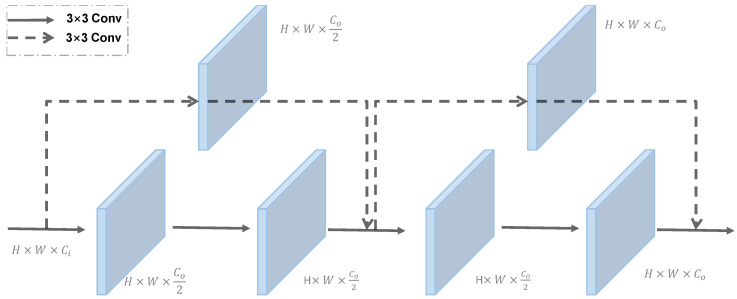
Structure of the dual-layer residual encoder. Dashed lines represent skip connections to enhance information propagation and learning. *H* and *W* represent input feature dimensions, *C* denotes the number of input channels, and $C^{\prime }$ refers to the number of output channels.

The dual-layer residual encoder module enables the network to learn shallow features and propagate them to deeper layers through residual connections, efficiently using and integrating feature information across different levels. The flowchart of the module is shown in Figure 2. This module consists of two convolutional blocks and two skip connection layers. Each convolutional block contains two convolutional layers, a batch normalization layer, and a ReLU activation function. This architecture is crucial for medical image segmentation tasks. Through feature reuse, it ensures the model extracts deep features while preserving shallow features, addressing the vanishing gradient problem and improving training efficiency.

Compared to models with single-layer residual structures [32,33], our dual-layer residual encoder module achieves deeper feature representations. Compared to models using DenseNet, VGG, or ResNet as encoders [34,35], our simpler architecture effectively preserves image detail features. This enables the encoder to capture context-aware regional feature representations, effectively bridging local fine-grained details with global long-range dependencies. It provides a robust feature foundation for subsequent patch-based graph inference, achieving hierarchical modeling from pixel-level locality to regional semantics.

### 3.2. Spatial-Channel Residual Dynamic Graph Convolution Feature Extraction Module

Due to the vanishing gradient problem, most advanced GCN models do not exceed four layers. Mitigating the vanishing gradient issue in GCNs has remained a persistent challenge. Fortunately, researchers have proposed promising solutions to this challenge. Li et al. leveraged residual connections and dilated convolutions from CNNs, demonstrating their significant contribution to constructing deep GCNs and mitigating the vanishing gradient problem [12]. Xuan et al. noted that, in traditional graph convolutions, the adjacency matrix remains static, which does not reflect the true dynamics of graph connectivity and topology. They proposed a dynamic graph convolution mechanism to capture the evolving state of graphs. This approach enhances segmentation models’ ability to define tumor integrity and identify edge details [36].

When processing image data, GCNs leverage the graph adjacency matrix to account for structural relationships between nodes. However, GCNs do not directly consider relative spatial positions between nodes, which is crucial in image data. To better fuse local and global information and improve model performance, we introduce positional encoding before the first node attribute matrix in the GCN. The position encoding matrix has the same dimensions as the node attribute matrix, introducing positional information for each location in the input features. Each element of the position encoding matrix corresponds to a positional encoding value at the same location as the node attribute matrix. By adding the position encoding matrix to the node attribute matrix, the model can use both node attribute information and positional information to better understand the global context.

**Figure 3:**
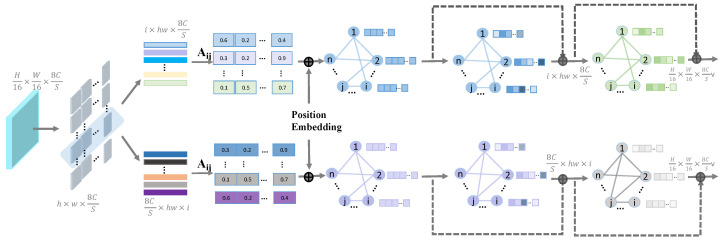
Dual-branch graph convolution module for spatial and channel features.

Based on these principles, we propose a dual-branch graph convolution module (see Figure 3). This module integrates residual connections, positional encoding, and dynamic graph convolutions to efficiently extract spatial and channel features from images, enhancing model performance.

### 3.3. Graph Construction

As shown in Figure 3, before inferring features through the GCN module, we must construct a graph representation from the encoder features. The feature update formula of GCN is as follows:

(1) $$F^{l+1}=\mathrm{GCN}\left(A^{l},F^{l},W^{l}\right)=\sigma \left(A^{l}F^{l}W^{l}\right)$$

where σ represents the activation function (ReLU), $F^{l}$ and $F^{l+1}$ represent the input and output of the *l*-th layer, respectively. $A^{l}$ is the adjacency matrix, and $W^{l}$ is the learnable weight matrix. Graph construction consists of two key components: node selection and edge definition. We construct the graph through the following two steps.

#### 3.3.1. Node Selection

Compared to natural images, medical image datasets are typically smaller. Using a Transformer to capture image context inevitably introduces many parameters, increasing the risk of overfitting. To address this issue, inspired by the node aggregation capabilities of GCNs, we replace the Transformer module with a GCN. However, most current GNN-based models for image segmentation only consider spatial relationships within and between channels, failing to fully integrate the Transformer’s patch-based approach to uncover long-range feature dependencies and preserve image details. In this work, we propose a combination of the Transformer’s patch-based approach with GCNs for medical image segmentation. Below we explain our node selection process.

Given the bottleneck feature map $F\in R^{C\times H\times W}$ from the encoder, we partition *F* into *S* non-overlapping spatial patches of size p×p, where $S=\frac{H}{p}\times \frac{W}{p}$. Each patch $F_{i}\in R^{C\times p\times p}$ is flattened to yield a node feature vector $x_{i}\in R^{C\times n}$, where n=p×p. Each flattened patch is treated as a node $V_{i}$ in the graph, so the node set is $V=\{V_{1},V_{2},\ldots ,V_{S}\}$ with |V|=S. This patch-based node definition groups spatially coherent encoder features into compact units, enabling graph convolution to model structured relationships between regions rather than individual pixels.

#### 3.3.2. Construction of the Adjacency Matrix

We adopt cosine similarity to construct edge weights, as it measures directional alignment between feature vectors independently of their magnitudes:

(2) $$A_{ij}=\frac{x_{i}\cdot x_{j}}{∥x_{i}∥∥x_{j}∥}$$

This choice is motivated by the characteristics of our experimental setting: (1) bottleneck features exhibit large magnitude variation across semantic regions (lesion vs. background), so direction-based similarity is more semantically meaningful than magnitude-sensitive Euclidean distance; (2) in the C=512-dimensional feature space, Euclidean distances suffer from concentration of measure, reducing discriminative power at high dimensionality, whereas cosine similarity operates on the unit hypersphere and remains informative; (3) cosine values in [−1, 1] become non-negative after ReLU suppression, directly satisfying the non-negativity requirement of spectral graph convolution without an additional kernel step [26].

The adjacency matrix is constructed as follows. For node pair (i,j), the raw similarity score is first computed as $A_{ij}=\frac{x_{i}\cdot x_{j}}{∥x_{i}∥∥x_{j}∥}$. To ensure non-negative edge weights (required for convolution stability), ReLU is applied to suppress negative similarities. Row-wise Softmax is then applied to normalize each row to sum to 1, yielding the final asymmetric adjacency matrix $A^{\prime }$. Self-connections are included by initializing diagonal entries to 1 before normalization. The resulting graph is fully connected (dense), with near-zero weights assigned to dissimilar node pairs through the competitive suppression of Softmax.

Based on the above process, we can construct a graph $G=(V,E,X,A^{\prime })$, where *V* and *E* represent the graph’s nodes and edges, *X* denotes the node attribute matrix, and $A^{\prime }$ represents the normalized adjacency matrix. However, while we introduced the node features $x_{i}$ in the previous subsection, we have not yet detailed how the node attribute matrix *X* is constructed based on $x_{i}$. In the following two subsections, we introduce the construction methods of the node attribute matrix from both spatial and channel dimensions and discuss the implementation process of each branch. Through this exploration, we gain a more comprehensive understanding of the node attribute matrix construction and the graph convolution mechanism implementation.

### 3.4. Dual-Branch Graph Inference Module

#### 3.4.1. Spatial Residual Dynamic Graph Convolution Feature Extraction Module

Unlike constructing spatial relationships using the HW×C node attribute matrix, our research focuses on the spatial correlation of individual image patches in corresponding channels. Dividing the overall feature into multiple feature patches allows us to capture local features and preserve spatial detail information while increasing the representation power of GCN’s local dimension and incorporating global dependencies. In this section, we capture global dependencies and preserve local details using the spatial node attribute matrix. The spatial approach is described as follows.

First, for the feature matrix $F\in R^{C\times H\times W}$ from the 2D encoder, we divide it into patches according to the specified patch size. We take the patches’ extracted spatial feature $F_{i}\in R^{C\times h\times w}$ as input and obtain the corresponding node features $x_{i}$ for each feature patch. To measure the spatial similarity relationship between each feature patch in the respective channels, we calculate $x_{i}$, $x_{j}$ on one channel to construct the spatial node attribute matrix $X_{s}$, $X_{s}\in R^{C\times i\times n}$. Here, *C* represents the number of channels, the second dimension *i* represents the number of feature patches, and the third dimension *n* represents the feature dimension of the patches. We denote the spatial node attribute matrix $X_{s}$ as $F_{s}$.

To help GCN distinguish the relative positions of different nodes and better capture local and global information in the graph structure, enhancing the model’s understanding and reasoning capabilities, we add positional encodings $P_{s}$ to $F_{s}$, where $P_{s}\in R^{C\times i\times n}$.

After obtaining the final spatial node attribute matrix, we construct the adjacency matrix $A_{si}$ for each channel according to the construction method described in Section 3.3, $A_{si}\in R^{i\times i}$. Then, we concatenate them along the channel dimension to obtain the final adjacency matrix $A_{s}$, $A_{s}\in R^{C\times i\times i}$.

Finally, after obtaining the spatial node attributes and corresponding adjacency matrix, we use a two-layer dynamic residual graph convolution to capture long-range dependencies in the image. The specific algorithm procedure is shown in Algorithm 1.
**Algorithm 1** Spatial Residual Dynamic Graph Convolutional Feature Extraction Module**Require:** K=2, *F*, *A***Ensure:** $F_{s}^{\prime }$ **Initialization:** **for** k=1 **to** *K* **do**  $W_{k}\leftarrow \mathrm{nn}.\mathrm{Parameter}\left(C,\;n,\;n\right)$ **end for** $P_{s}\leftarrow \mathrm{nn}.\mathrm{Parameter}\left(C,\;i,\;n\right)$ **Preparation for GCN:** $F_{s}\leftarrow \mathrm{reshape}\left(F\right)$ $F_{s}\leftarrow F_{s}+P_{s}$ **Graph Convolution Loop:** $F_{s}^{\left(0\right)}\leftarrow F_{s}$ **for** k=1 **to** *K* **do**  $A^{\left(k\right)}\leftarrow \mathrm{adjacency}\left(F_{s}^{\left(k-1\right)}\right)$  $A^{\left(k\right)}\leftarrow \mathrm{Softmax}\left(A^{\left(k\right)},\;\dim=-1\right)$  $F_{s}^{\left(k\right)}\leftarrow \mathrm{GCN}\left(A^{\left(k\right)},\;F_{s}^{\left(k-1\right)},\;W_{k}\right)$$\left\{F_{s}^{\left(k\right)}=\sigma \;\left(A^{\left(k\right)}F_{s}^{\left(k-1\right)}W_{k}\right)\right\}$  $F_{s}^{\left(k\right)}\leftarrow F_{s}^{\left(k\right)}+F_{s}^{\left(k-1\right)}$  $F_{s}^{\left(k\right)}\leftarrow \mathrm{ReLU}\left(F_{s}^{\left(k\right)}\right)$ **end for** $F_{s}^{\prime }\leftarrow F_{s}^{\left(K\right)}$ **Preparation for CNN:** $F_{s}^{\prime }\leftarrow \mathrm{reshape}\left(F_{s}^{\prime }\right)$ **return** $F_{s}^{\prime }$

Through this approach, we can more accurately capture global dependencies in images while preserving local details, enhancing the model’s performance and reasoning capabilities.

#### 3.4.2. Channel Residual Dynamic Graph Convolution Feature Extraction Module

Unlike approaches that use the C×HW node property matrix to account for inter-channel relationships, we propose that the channel relationships corresponding to each feature patch are inherently distinct. Considering only overall correlations among feature channels may compromise effective extraction of channel information. We introduce the concept of incorporating channel correlations between individual feature patches while ultimately integrating relationships across all feature patch channels. We believe this approach embodies aspects of multi-head attention, potentially enhancing the model’s generalization performance. The specific implementation is described below.

Similar to the spatial graph convolution branch, this module primarily considers channel-level relationships within each feature patch. To achieve this, we modify the spatial node attribute matrix $X_{s}$. As indicated in Section 3.4.1, $X_{s}\in R^{C\times i\times n}$. To leverage GCNs for capturing relationships between channels within each feature patch, we transpose $X_{s}$ to obtain $X_{c}\in R^{i\times C\times n}$, constructing a channel node attribute matrix. Here, *i* of $X_{c}$ represents the number of feature patches, *C* represents the number of channels, and *n* represents the feature dimension of the patch. Through this adjustment, we can analyze channel relationships within each patch. We denote the channel node attribute matrix $X_{c}$ as $F_{c}$.

To better capture channel features, following the method in Section 3.4.1, we add positional encodings $P_{c}$ to $F_{c}$, where $P_{c}\in R^{i\times C\times n}$. Then, based on $X_{c}$, we construct the adjacency matrix $A_{ci}$ for each patch according to the construction method in Section 3.3, where $A_{ci}\in R^{C\times C}$. Finally, by concatenating along the patch dimension, we obtain the final adjacency matrix $A_{c}$, where $A_{c}\in R^{i\times C\times C}$. After obtaining the node attribute features on the channel and the corresponding adjacency matrix, we use a two-layer dynamic residual graph convolution to capture long-range dependency relationships in the image. The specific algorithm flowchart is shown in Algorithm 2.
**Algorithm 2** Channel Residual Dynamic Graph Convolutional Feature Extraction Module**Require:** K=2, *F***Ensure:** $F_{c}^{\prime }$ **Initialization:** **for** k=1 **to** *K* **do**  $W_{k}\leftarrow \mathrm{nn}.\mathrm{Parameter}\left(i,\;n,\;n\right)$ **end for** $P_{c}\leftarrow \mathrm{nn}.\mathrm{Parameter}\left(i,\;C,\;n\right)$ **Preparation for GCN:** $F_{c}\leftarrow \mathrm{reshape}\left(F\right)$ $F_{c}\leftarrow F_{c}+P_{c}$ **Graph Convolution Loop:** $F_{c}^{\left(0\right)}\leftarrow F_{c}$ **for** k=1 **to** *K* **do**  $A^{\left(k\right)}\leftarrow \mathrm{adjacency}\left(F_{c}^{\left(k-1\right)}\right)$  $A^{\left(k\right)}\leftarrow \mathrm{Softmax}\left(A^{\left(k\right)},\;\dim=-1\right)$  $F_{c}^{\left(k\right)}\leftarrow \mathrm{GCN}\left(A^{\left(k\right)},\;F_{c}^{\left(k-1\right)},\;W_{k}\right)$$\left\{F_{c}^{\left(k\right)}=\sigma \;\left(A^{\left(k\right)}F_{c}^{\left(k-1\right)}W_{k}\right)\right\}$  $F_{c}^{\left(k\right)}\leftarrow F_{c}^{\left(k\right)}+F_{c}^{\left(k-1\right)}$  $F_{c}^{\left(k\right)}\leftarrow \mathrm{ReLU}\left(F_{c}^{\left(k\right)}\right)$ **end for** $F_{c}^{\prime }\leftarrow F_{c}^{\left(K\right)}$ **Preparation for CNN:** $F_{c}^{\prime }\leftarrow \mathrm{reshape}\left(F_{c}^{\prime }\right)$ **return** $F_{c}^{\prime }$

By performing message passing on the channel map through dual-layer dynamic residual graph convolutions, this module adaptively aggregates cross-channel contextual information, enhances channel responses that are discriminative for lesion regions, and suppresses interference from noise and irrelevant channels. This region-specific, channel-specific fine-grained modeling approach complements the spatial module’s cross-region modeling. Together, they establish a comprehensive representation of region-level feature consistency and inter-region relational structure, significantly enhancing the model’s robustness in segmenting low-contrast, weakly-defined small targets.

### 3.5. SC-GCN Integration Module

**Figure 4:**
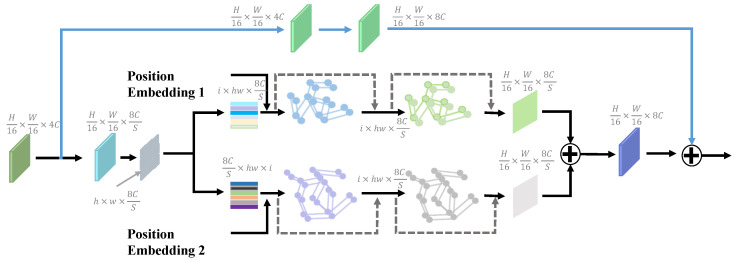
Detailed structure of the SC-GCN integration module.

In Section 3.4, we detailed the Spatial-Channel Graph Convolutional network constructed using GCNs to capture global dependencies in images. However, based on the adjacency matrix definition in Section 3.3, using too many channels in the graph convolution module for spatial-channel data would result in prohibitively large computational costs for the adjacency matrix operations in channel graph convolutions. To better fuse channel features while reducing the computational load of the adjacency matrix in graph convolutions, we apply a 3 × 3 convolution operation to the features before they enter the graph convolution module. This reduces the number of channels from 512 to 512/*r*, where *r* is a channel reduction ratio. To better preserve fine-grained information, we incorporate a residual connection. This connection consists of two 3 × 3 CNN convolutions, a batch normalization function, and a ReLU activation function. The detailed SC-GCN module is shown in Figure 4.

### 3.6. Loss Function

We use the commonly used BCE-Dice Loss as the loss function for each decoder branch, summing the losses across all branches to obtain the final loss function. The specific mathematical expression is shown below.

(3) $$\begin{array}{c} L_{\mathrm{bce}}^{\left(l\right)}=-\frac{1}{N}\sum_{n=1}^{N}\left[y_{n}\log\left(p_{n}^{\left(l\right)}\right)+\left(1-y_{n}\right)\log\left(1-p_{n}^{\left(l\right)}\right)\right] \end{array}$$

(4) $$\begin{array}{c} L_{\mathrm{dice}}^{\left(l\right)}=1-\frac{2\sum_{n=1}^{N}y_{n}p_{n}^{\left(l\right)}+\varepsilon }{\sum_{n=1}^{N}y_{n}+\sum_{n=1}^{N}p_{n}^{\left(l\right)}+\varepsilon } \end{array}$$

(5) $$\begin{array}{c} L^{\left(l\right)}=L_{\mathrm{bce}}^{\left(l\right)}+L_{\mathrm{dice}}^{\left(l\right)} \end{array}$$

(6) $$\begin{array}{c} \mathrm{Loss}=\sum_{l=1}^{5}L^{\left(l\right)} \end{array}$$

where *l* is the decoder layer index, *N* represents the total number of image pixels, $y_{n}$ is the ground-truth label, and $p_{n}^{\left(l\right)}$ is the predicted value at layer *l*. All five decoder branches are weighted equally with coefficient 1.0. No layer-specific weighting is applied, consistent with standard deep supervision practice.

## 4. Experiments

### 4.1. Dataset Description

All experiments were conducted on three publicly available datasets: Kvasir-SEG [37], BUSI [38], and COVID-19 CT [39].

#### 4.1.1. Kvasir-SEG

The Kvasir-SEG dataset supports research on colonic polyp segmentation, detection, localization, and classification. The dataset includes 1000 colonic polyp images with their corresponding ground-truth annotations from the Kvasir dataset v2. The images have varying resolutions, ranging from 332 × 487 to 1920 × 1072 pixels. Both images and their corresponding masks are stored in separate folders with identical file names.

#### 4.1.2. BUSI

The BUSI (Breast Ultrasound Image) dataset contains breast ultrasound images collected in 2018 from 600 female patients aged between 25 and 75 years. The dataset originally consists of 780 images (437 benign, 210 malignant, and 133 normal) with an average size of 500 × 500 pixels. The dataset includes detailed segmentation annotations for benign and malignant cases, providing crucial information for precise lesion delineation. In our experiments, we restrict evaluation to the 647 images containing confirmed lesions (437 benign and 210 malignant). The 133 normal images, which carry no foreground lesion masks and thus provide no segmentation supervision signal, are excluded. This follows standard practice in lesion segmentation benchmarking, where the evaluation objective is boundary delineation of pathological regions rather than disease screening.

#### 4.1.3. COVID-19 CT

A large lung CT scan dataset for COVID-19 was constructed by curating data from seven public datasets. Three of them contained shared COVID-19 lesion masks. This dataset combines the COVID-19 lesion masks and their corresponding frames from these three public datasets, resulting in 2729 image and ground-truth mask pairs. To ensure consistency across datasets, all different types of lesions are mapped to white color.

### 4.2. Data Processing

For the Kvasir-SEG and BUSI datasets, we applied random image rotation data augmentation within a range of −10° to 10°. Since the COVID-19 CT dataset already contained sufficient data, no data augmentation was performed. After augmentation, the Kvasir-SEG dataset had 2000 samples, and the BUSI dataset had 1294 samples. The COVID-19 CT dataset remained at 2729 samples without augmentation. All images and corresponding masks are resized to 192 × 192 via isotropic bilinear interpolation, followed by normalization.

### 4.3. Experimental Settings

All experiments were conducted using PyTorch 2.4.1 with the Adam optimizer on NVIDIA Quadro RTX 6000 (24 GB) and NVIDIA GeForce RTX 2060 SUPER (8 GB) GPUs.

For dataset splitting, the BUSI dataset was divided into 70% training, 20% validation, and 10% testing sets. Splits are performed with stratification by pathological category (benign versus malignant), ensuring that each subset maintains approximately the same proportion of benign and malignant cases as the full 1294-image cohort, thereby preventing performance estimation bias toward either lesion type. Experiments were repeated three times using different random seeds to ensure reliability. The Kvasir-SEG and COVID-19 CT datasets employed 5-fold cross-validation for comprehensive performance evaluation.

The hyperparameters were configured as follows: weight decay of 0.0001, initial learning rate of 0.0001, and batch size of 4. The model was trained for a maximum of 90 epochs with the learning rate decayed by a factor of 0.1 every 15 epochs; early stopping with a patience of 20 epochs was applied. For the graph module, the patch size *p* was set to 4, resulting in S=9 nodes on the 12 × 12 bottleneck feature map (corresponding to an input resolution of 192 × 192). The channel reduction ratio *r* in the SC-GCN integration module was set to 32, compressing the channel dimension from 512 to 16 before graph convolutions. All convolutional layers use Kaiming normal initialization with fan-in mode; GCN weight matrices use Xavier uniform initialization; all bias terms are initialized to zero. The model checkpoint with the lowest validation loss is saved and used for final testing. For BUSI, experiments are repeated with random seeds {42, 123, 2024}; for 5-fold cross-validation datasets, seed 42 is fixed within each fold. No explicit class-weighting or oversampling is applied; the BCE-Dice joint loss provides inherent robustness to class imbalance through its Dice component. For BUSI, 20% of the training split is held out as validation; for 5-fold cross-validation datasets, 20% of each training fold serves as validation for scheduling and early stopping.

### 4.4. Evaluation Metrics

To conduct a comprehensive analysis of the differences among models, we used a range of metrics to assess their performance. These metrics included Recall (Rec), Precision (Prec), Accuracy (Acc), Specificity (SP), Dice Similarity Coefficient (DSC), F2 score (F2), Intersection over Union (IOU), Mean IOU (MIOU), and Hausdorff Distance (HD). The calculation formulas are defined as follows:

(7) $$\begin{array}{cc} \mathrm{Rec} & =\frac{TP}{TP+FN} \end{array}$$

(8) $$\begin{array}{cc} \mathrm{Prec} & =\frac{TP}{TP+FP} \end{array}$$

(9) $$\begin{array}{cc} \mathrm{ACC} & =\frac{TP+TN}{TP+FP+FN+TN} \end{array}$$

(10) $$\begin{array}{cc} \mathrm{SP} & =\frac{TN}{TN+FP} \end{array}$$

(11) $$\begin{array}{cc} \mathrm{DSC} & =\frac{2TP}{2TP+FP+FN} \end{array}$$

(12) $$\begin{array}{cc} F2 & =\frac{5\times \mathrm{Precision}\times \mathrm{Recall}}{4\times \mathrm{Precision}+\mathrm{Recall}} \end{array}$$

(13) $$\begin{array}{cc} {\mathrm{IOU}}_{\mathrm{label}} & =\frac{TP}{TP+FP+FN} \end{array}$$

(14) $$\begin{array}{cc} {\mathrm{IOU}}_{\mathrm{background}} & =\frac{TN}{TN+FN+FP} \end{array}$$

(15) $$\begin{array}{cc} \mathrm{MIOU} & =\frac{1}{2}\times \left({\mathrm{IOU}}_{\mathrm{label}}+{\mathrm{IOU}}_{\mathrm{background}}\right) \end{array}$$

(16) $$\begin{array}{cc} \mathrm{HD} & =\max\left\{\underset{a\in A}{\sup}\underset{b\in B}{\inf}d\left(a,b\right),\underset{b\in B}{\sup}\underset{a\in A}{\inf}d\left(a,b\right)\right\} \end{array}$$

where TP, FP, TN, and FN denote true-positive, false-positive, true-negative, and false-negative samples, respectively. In Equation (16), *A* and *B* represent the boundary point sets of the predicted segmentation and ground-truth mask, respectively, while d(·,·) denotes the Euclidean distance. Hausdorff Distance measures the maximum boundary discrepancy between two sets, with lower values indicating better segmentation accuracy.

### 4.5. Comparison Methods

To comprehensively evaluate the performance of our proposed SCAGC-UNet, we compare it against nine state-of-the-art medical image segmentation methods representing different architectural paradigms.

CNN-based architectures: UNet is the foundational encoder–decoder network. ACC-UNet and CCBANet enhance UNet with attention mechanisms to capture multi-scale contextual features.

Transformer-based architectures: TransUNet combines CNNs with Transformers for global context modeling. SwinUNet employs shifted window self-attention for hierarchical feature learning. CMUNeXt integrates convolutional and multi-scale Transformer modules.

Hybrid architectures: UNext utilizes modern depthwise separable convolutions with reduced parameters. MK-UNet employs multi-kernel convolutions for multi-scale feature extraction. EMCAD adopts ensemble strategies to improve segmentation robustness.

All baseline models were implemented using official code repositories with their recommended hyperparameters. To ensure a fair architectural comparison, all methods share identical preprocessing (isotropic bilinear resize to 192 × 192, followed by normalization), data splits, batch size, optimizer (Adam, lr = 0.0001, weight decay = 0.0001), learning rate schedule, early stopping patience (20 epochs), augmentation strategy, and evaluation metrics. For methods with officially recommended configurations that differ from our defaults (e.g., SwinUNet’s ImageNet-21K pre-trained weights), these are applied as recommended by the original authors.

### 4.6. Quantitative Results

**Figure 5:**
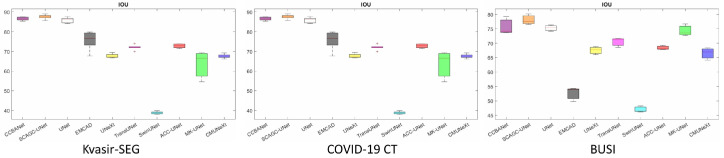
Box plot analysis of IOU performance across three datasets. SCAGC-UNet (orange) consistently achieves the highest median values and lowest variance, demonstrating superior performance and stability.

We evaluate the performance of SCAGC-UNet against nine state-of-the-art baseline methods across three medical image segmentation datasets. The quantitative results are presented in Table 1, Table 2 and Table 3, while Figure 5 displays box plots of IOU distributions to visualize performance stability across all methods. The box plot analysis reveals three consistent observations. First, SCAGC-UNet achieves the highest median IOU across all three datasets (BUSI: 78%, COVID-19 CT: 70.5%, Kvasir-SEG: 87.5%). Second, the narrow interquartile range of SCAGC-UNet indicates exceptionally low performance variability, in contrast to methods such as ACC-UNet and MK-UNet which exhibit large variance. Third, our model consistently maintains high lower bounds (25th percentile), ensuring robust minimum performance even in challenging cases—particularly on the BUSI dataset where our lower bound (77%) exceeds the median of most competing methods.

#### 4.6.1. Results on Kvasir-SEG Dataset

Table 1 presents the quantitative results on the Kvasir-SEG polyp segmentation dataset. Our model achieves the highest performance across most metrics, with an IOU of 87.50% and MIOU of 92.41%. Compared to the second-best model CCBANet, our approach yields improvements of 1.03% in IOU and 0.64% in MIOU, while surpassing the classical UNet by 1.60% and 1.03%, respectively. More notably, our method demonstrates substantial advantages over recent architectures: 11.68% improvement over EMCAD (IOU 75.82%), 19.74% over MK-UNet (IOU 67.76%), and gains of 48.59% and 15.48% over Transformer-based methods SwinUNet (IOU 38.91%) and TransUNet (IOU 72.02%), respectively. Notably, the low performance of SwinUNet (IOU 38.91%) is consistent with the broader pattern of performance degradation observed across Transformer-based methods on small-lesion benchmarks. Two core factors lead to this phenomenon: a domain distribution gap, since SwinUNet is originally developed for abdominal multi-organ CT segmentation, which differs substantially from the polyp endoscopic and breast ultrasound imaging tasks adopted in this work, and inherent architectural sensitivity to small lesions. Early global window-attention aggregation tends to dilute discriminative local boundary features when lesion regions only occupy a tiny fraction of the entire image. This performance pattern aligns with the well-recognized limitations of global attention aggregation in medical imaging research, and is further corroborated by the performance gap between TransUNet and CNN-based UNet on the BUSI dataset (IOU 70.50% vs. UNet 75.28%).

#### 4.6.2. Results on COVID-19 CT Dataset

As shown in Table 2, SCAGC-UNet achieves substantial performance gains on the COVID-19 CT segmentation task, attaining a lesion region IOU of 70.23% and MIOU of 85.87%. These metrics outperform UNet (IOU 66.10%, MIOU 82.79%) by 4.13% and 3.08%, respectively. Compared to attention-enhanced methods CCBANet (IOU 62.83%), and recent architectures MK-UNet (IOU 63.44%) and EMCAD (IOU 64.47%), our approach achieves IOU improvements of 7.40%, 6.79%, and 5.76%. The advantages over Transformer-based methods are even more pronounced, with gains of 6.75% over TransUNet and 15.56% over SwinUNet. These results are consistent with the architectural analysis presented in the Kvasir-SEG discussion: Transformer-based methods exhibit reduced performance on datasets with small, low-contrast lesions, while SCAGC-UNet’s explicit region-level graph reasoning provides complementary advantages by modeling inter-region topological structure and intra-region channel semantics that global attention mechanisms do not capture through uniform spatial aggregation.

#### 4.6.3. Results on BUSI Dataset

Table 3 presents results on the BUSI breast ultrasound segmentation dataset, where SCAGC-UNet achieves an IOU of 78.10% and MIOU of 87.68%, outperforming all competing methods. Specifically, our model surpasses the second-best CCBANet (IOU 72.86%) by 5.24% in IOU, and exceeds UNet (75.28%) and MK-UNet (68.40%) by 2.82% and 9.70%, respectively. These improvements are particularly significant given the challenging characteristics of ultrasound imaging, including low contrast, ambiguous boundaries, and speckle noise. Furthermore, our model achieves an optimal balance between Recall (87.07%) and Precision (86.60%), indicating robust detection capability without excessive false-positives. The consistent performance gain on this dataset demonstrates that the channel branch graph module effectively mines regional semantic consistency, enhancing discrimination of lesions in noisy, low-contrast environments.

The model consistently achieves superior segmentation performance across three distinct imaging modalities (endoscopy, CT and ultrasound) as well as lesions with diverse characteristics. These results demonstrate that, under a unified evaluation protocol, SCAGC-UNet can deliver stable and reliable segmentation performance on public benchmark datasets from various imaging domains. The experimental results presented only indicate that this method has potential clinical reference value. By explicitly modeling region-level features through dual-branch graph convolutions, our framework effectively bridges the semantic gap between pixel-level locality and global long-range dependencies, enabling robust feature aggregation across different imaging conditions and anatomical structures.

## 5. Discussion

### 5.1. Qualitative Analysis

**Figure 6:**
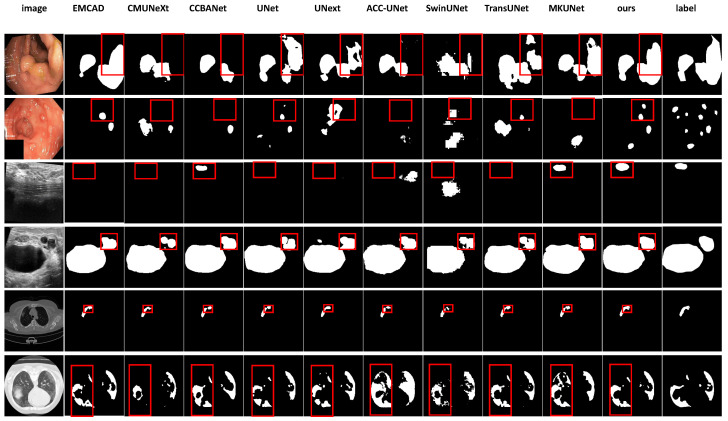
Qualitative comparison of segmentation results across different methods. The first two rows show results from the Kvasir-SEG dataset, the middle two rows show the BUSI dataset, and the last two rows show the COVID-19 CT dataset. SCAGC-UNet consistently produces more accurate segmentations with better boundary delineation.

To illustrate our model’s performance, Figure 6 presents qualitative segmentation results on representative samples from the three datasets. (The selected samples cover lesions of different sizes, morphologies and image qualities, instead of deliberately choosing cases with optimal segmentation results.) On the Kvasir-SEG dataset (rows 1–2), SCAGC-UNet produces more complete polyp delineations with tighter boundary adherence, even in cases where lesion edges are inherently blurry and poorly contrasted against the surrounding mucosa. On the BUSI dataset (rows 3–4), our model demonstrates superior preservation of lesion contours under low-contrast and speckle-corrupted conditions, where several competing methods either over-segment the background or miss fine boundary structures. On the COVID-19 CT dataset (rows 5–6), SCAGC-UNet more accurately localizes diffuse lesion regions with irregular morphology, exhibiting fewer false-positives in non-lesion areas compared to both CNN-based and Transformer-based baselines. Collectively, these qualitative results corroborate the quantitative findings, confirming that the dual-branch graph convolutional design effectively integrates global contextual information with local boundary details across diverse imaging modalities.

In addition, we further compare the parameter counts and Flops of all models (Table 4). SCAGC-UNet achieves competitive segmentation accuracy among the evaluated methods while requiring only 155.85 G Flops and 16.74 M parameters, demonstrating a favorable accuracy–efficiency trade-off. In terms of Flops, SCAGC-UNet uses substantially fewer operations than UNet (589.02 G, a 3.78× reduction) and ACC-UNet (532.63 G, a 3.42× reduction), both of which yield considerably lower segmentation accuracy. Among the high-accuracy methods (DSC > 90% on Kvasir-SEG)—only CCBANet and UNet besides SCAGC-UNet—our method requires only 26.5% of UNet’s Flops while achieving 1.19% higher DSC on Kvasir-SEG and 2.82% higher IoU on BUSI. Compared with CCBANet (7.80 G), SCAGC-UNet incurs moderately more computation (20.0× higher Flops), yet delivers 0.73% higher DSC on Kvasir-SEG and 5.24% higher IoU on BUSI—a favorable accuracy gain relative to the added computational cost. Regarding parameters, SCAGC-UNet contains only 16.74 M parameters, fewer than CCBANet (31.57 M), UNet (34.53 M), SwinUNet (41.37 M), TransUNet (105.32 M), and MK-UNet (315.57 M). Notably, ACC-UNet has nearly the same parameter count (16.77 M) as SCAGC-UNet, yet achieves 10.88% lower DSC on Kvasir-SEG and 9.71% lower IoU on BUSI, suggesting that the performance advantage of SCAGC-UNet is likely due to its architectural design rather than parameter scale. These results demonstrate that the SC-GCN module enables efficient global region reasoning without imposing prohibitive computational overhead.

### 5.2. Ablation Study

#### 5.2.1. Component Ablation

To evaluate the contribution of each proposed module, we conducted ablation experiments on all three datasets by progressively incorporating components into the baseline model. The baseline model (RD) employs a standard encoder without any dynamic graph convolution modules. Building upon RD, we constructed two variants: RDC, which integrates only the channel graph convolution branch, and RDS, which integrates only the spatial graph convolution branch; the ablation results are presented in Table 5.

As shown in Table 5, the contribution of the SC-GCN module is strongly dataset-dependent, reflecting the degree of structural heterogeneity in each imaging modality. On Kvasir-SEG, where polyp morphology is relatively uniform and the RD baseline already achieves 92.28% MIOU, the incremental improvements from adding graph convolution branches are modest (RD→Full: +0.13 pp MIOU, HD 15.98→15.32 mm). This near-saturated performance ceiling is expected and reflects the limited room for improvement on this benchmark, rather than a limitation of the SC-GCN module itself. In contrast, on the more structurally heterogeneous COVID-19 CT dataset, the SC-GCN module provides substantial gains: the full model achieves MIOU 85.87%, compared to 74.10% for the RD baseline (+11.77 pp), with HD improving dramatically from 37.69mm to 18.64mm (a 50.6% reduction). Similarly, on BUSI, the full model achieves MIOU 87.68% versus 81.57% for RD (+6.11 pp), with HD improving from 17.49 mm to 14.87 mm (14.9% reduction).

RDC achieves consistent performance gains over RD across all three datasets, demonstrating the effectiveness of the channel graph convolution branch. Notably, within the narrow performance range available near the Kvasir-SEG ceiling, RDC yields the most substantial boundary localization improvement: Hausdorff Distance decreases from 15.98 mm (RD) to 14.29 mm (RDC), a 1.69 mm reduction that halves the discrepancy margin available for improvement relative to the best-performing model. This demonstrates that channel-level semantic consistency modeling contributes meaningfully to boundary precision even when aggregate overlap metrics show diminishing returns—a property particularly relevant in boundary-sensitive clinical applications. By contrast, the spatial branch alone (RDS, HD 15.81 mm) does not replicate this boundary gain when operating in isolation, confirming that it is intra-region channel consistency, rather than inter-region spatial topology alone, that drives boundary precision on this near-saturated benchmark.

Notably, the full model incorporating both branches outperforms all individual variants across all three datasets, confirming that the channel and spatial graph convolution branches act synergistically rather than competitively, and their combination yields complementary feature representations that benefit both segmentation overlap and boundary localization.

#### 5.2.2. Encoder Backbone Ablation

To assess the effectiveness of the proposed dual-layer residual encoder, we replaced it with two alternative encoders—the standard UNet encoder and the ResNet34 encoder—while keeping the spatial and channel graph convolution modules unchanged, yielding models UNetR and ResR, respectively. As reported in Table 6, both UNetR and ResR exhibit inferior performance compared to the full model across all three datasets, confirming that the dual-layer residual encoder contributes meaningfully to the overall segmentation accuracy.

On Kvasir-SEG, a notable observation is that ResR achieves the highest Recall (93.20%) among all encoder variants, while SCAGC-UNet achieves higher Precision (94.16%), Specificity (99.09%), DSC (92.28%), IOU (87.50%), and MIOU (92.41%). This pattern indicates that ResR tends toward more aggressive boundary expansion—detecting more foreground at the cost of increased false-positives—whereas SCAGC-UNet, equipped with the dual-layer residual encoder, produces tighter and more precise boundary delineation. This is further corroborated by the HD metric: SCAGC-UNet achieves HD 14.32 mm, lower than ResR (14.93 mm) and UNetR (15.89 mm), confirming superior boundary localization accuracy. On BUSI, SCAGC-UNet achieves the best IOU (78.10%) and the lowest HD (14.87 mm) despite UNetR showing higher DSC (86.42%), indicating that the dual-layer residual encoder contributes to more precise boundary adherence even when overall segmentation overlap is comparable.

**Figure 7:**
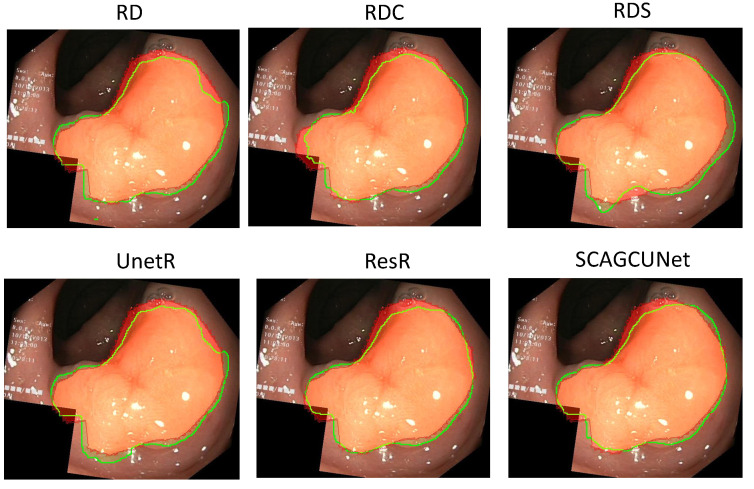
Qualitative segmentation results of ablation model variants on the Kvasir-SEG dataset. Green and red contours indicate the predicted boundary and ground-truth, respectively.

To further qualitatively assess the impact of each module, Figure 7 presents representative segmentation results produced by all ablation variants on the Kvasir-SEG dataset. The green contour denotes the predicted segmentation boundary, while the red contour represents the ground-truth. The visual observations are consistent with the quantitative metrics in Table 5. RD exhibits the largest boundary deviations from the ground-truth, with noticeable over-segmentation in background regions and loose contour adherence—consistent with its lowest Precision (93.80%) and highest HD (15.98 mm) among all variants. RDC produces the tightest boundary adherence in terms of physical contour proximity, reflecting its lowest HD (14.29 mm) and highest Recall (92.48%), though its slightly lower IOU compared to the full model indicates that boundary proximity alone does not fully capture segmentation completeness. RDS, despite incorporating spatial structure, shows conservative and occasionally fragmented boundary predictions, with the contours tending to under-segment lesion regions—consistent with its highest Precision (94.04%) paired with the lowest DSC (92.12%) and Recall (92.07%), suggesting that the spatial branch operating in isolation produces overly conservative predictions that miss fine boundary details. The proposed full model, SCAGC-UNet, achieves the best overall segmentation fidelity: its predicted contours demonstrate the highest boundary precision (Prec 94.16%, SP 99.09%) with the fewest false-positive regions, and the highest area overlap (IOU 87.50%, MIOU 92.41%), demonstrating that the integration of both graph convolution branches yields complementary boundary representations—the channel branch contributing to fine-grained boundary localization and the spatial branch providing topological structural consistency—whose combination is superior to either branch operating independently.

**Figure 8:**
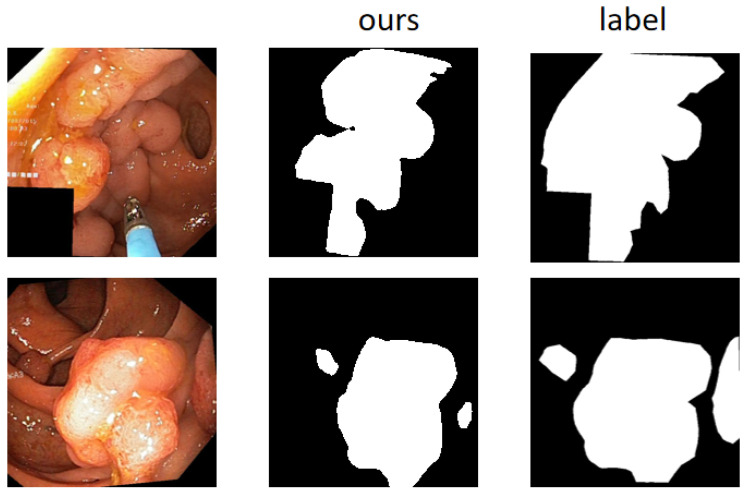
Representative failure cases of SCAGC-UNet on the Kvasir-SEG dataset.

Despite the promising results, several limitations of the current work should be acknowledged. The patch size used in the spatial graph convolution branch is a task-specific hyperparameter that requires manual tuning, which may limit the generalizability of the spatial branch across datasets with substantially different lesion scales. Figure 8 illustrates representative failure cases on the Kvasir-SEG dataset: the model exhibits under-segmentation on polyps with flat morphology and indistinct boundaries, where fixed-size patch partitioning causes region nodes to aggregate mixed lesion-background features, resulting in incomplete lesion delineation. This failure mode is directly linked to the patch size sensitivity of the spatial branch—when lesion boundary transitions occur within a single patch, the region-level graph cannot resolve the boundary at sub-patch granularity. More broadly, the graph hyperparameters of SCAGC-UNet—including the number of region nodes *S*, the GCN depth *K*, and the patch size—were configured based on theoretical considerations and applied uniformly across all datasets; a systematic empirical sensitivity analysis remains an acknowledged limitation. In addition, the current framework operates on 2D image slices, and extension to volumetric 3D data remains an open challenge. In principle, the SC-GCN module can be adapted for 3D data by constructing inter-slice edges connecting corresponding region nodes across adjacent slices (slice *z* and z±1), with edge weights determined by inter-slice cosine similarity—effectively extending the 2D region graph to a volumetric graph with S×D nodes (where *D* is the slice depth). Practical implementation would require sparse inter-slice connectivity and memory-efficient chunking strategies to manage the increased computational overhead. Furthermore, while SCAGC-UNet demonstrates consistent performance across three imaging modalities, all evaluations are conducted on public benchmark datasets; generalization to institution-specific data, rare pathology subtypes, and multi-center acquisition protocols remains unvalidated. The reported results support the *potential clinical relevance* of the proposed approach but do not constitute evidence of clinical utility in deployed settings, and prospective external validation is required before any clinical translation.

Future work will pursue several directions. We plan to investigate adaptive patch partitioning strategies that automatically determine semantically optimal graph node granularity without manual tuning. A systematic hyperparameter sensitivity analysis—evaluating patch size p∈{2,3,4,6} (corresponding to node counts S∈{36,16,9,4} on the 12 × 12 bottleneck feature map), GCN depth K∈{1,2,3}, and the channel reduction ratio *r*—is planned to provide rigorous empirical guidance for practitioners adapting SCAGC-UNet to new imaging contexts. The integration of graph inference with self-attention mechanisms in a deeper synergistic manner represents another promising avenue, potentially enabling complementary modeling of local topology and global context within a unified framework. Furthermore, extending SCAGC-UNet to 3D medical image segmentation represents a priority direction: the proposed SC-GCN can be extended by constructing a volumetric region graph in which intra-slice spatial edges are complemented by inter-slice edges connecting region nodes across adjacent axial planes, enabling joint modeling of within-slice topology and cross-slice contextual dependencies. Multimodal fusion scenarios—such as combining CT and PET modalities—would further broaden its applicability in complex clinical workflows and multi-source diagnostic tasks.

## 6. Conclusions

This paper presents SCAGC-UNet, a region-aware graph inference framework for medical image segmentation. By explicitly defining encoder-derived spatial partitions with high intra-region feature consistency as graph nodes, our approach models an intermediate semantic level that existing CNN and Transformer architectures overlook—bridging pixel-level local detail with image-level global context. The dual-layer residual encoder provides stable hierarchical feature extraction, while the dual-branch Spatial-Channel Graph Convolution module captures inter-region topological relationships and intra-region channel semantic consistency in a unified and complementary manner. Experiments across three public datasets spanning distinct imaging modalities—endoscopic polyp segmentation, breast ultrasound segmentation, and COVID-19 CT lesion segmentation—demonstrate consistent state-of-the-art performance, validating both the effectiveness and cross-modal generalization of the proposed framework. Notwithstanding these results, several limitations should be noted. The model exhibits sensitivity in scenarios involving ambiguous or diffuse lesion boundaries, where the spatial graph branch operating in isolation underperforms compared to the full model; performance on small deeply embedded lesions in BUSI remains below that of some encoder variants in terms of Recall. Furthermore, all evaluations are conducted on public benchmark datasets, and the results represent *potential clinical relevance* rather than demonstrated clinical utility; prospective multi-center validation is required before clinical translation. These results underscore the value of explicit region-level modeling in addressing the dual limitations of local CNN receptive fields and global Transformer semantic dilution, and establish graph-based region reasoning as a principled and effective paradigm for medical image segmentation that warrants further validation in real-world clinical settings.

## Figures and Tables

**Table 1:** Performance comparison of different models on the Kvasir-SEG dataset.

Method	Rec	SP	Prec	DSC	F2	ACC	IOU_label_	IOU_bg_	MIOU	HD
EMCAD [40]	90.84±2.02	93.35±5.46	81.87±6.13	86.25±4.03	86.06±2.42	93.30±4.30	75.82±4.57	83.71±5.12	83.71±4.81	44.66±3.47
CMUNeXt [41]	77.56±1.83	97.35±2.32	82.20±0.39	75.53±0.50	75.74±0.59	93.57±0.39	66.10±0.39	92.48±2.05	79.29±0.66	28.35±2.75
CCBANet [30]	92.75±0.54	98.56±0.13	92.18±0.82	91.55±0.72	91.89±0.66	97.68±0.17	86.47±1.03	97.07±0.21	91.77±0.61	16.66±0.93
UNet [1]	91.43±0.73	98.89±0.08	93.41±0.68	91.09±0.86	90.98±0.82	97.55±0.17	85.90±1.16	96.87±0.21	91.38±0.67	17.82±1.13
UNeXt [42]	75.99±0.89	98.32±0.10	86.64±1.01	77.23±0.99	75.83±0.95	94.21±0.22	67.76±1.24	93.20±0.29	80.48±0.73	28.18±1.31
ACC-UNet [43]	83.04±1.00	97.46±0.18	85.67±1.13	81.40±1.08	81.80±1.03	95.20±0.26	72.85±1.46	94.22±0.29	83.48±0.87	24.20±2.06
Swin-UNet [44]	60.09±3.94	93.09±0.69	56.12±1.40	51.64±1.71	54.28±2.64	69.81±0.25	38.91±1.53	85.72±0.30	62.31±0.76	63.35±2.18
TransUNet [45]	82.14±0.99	97.71±0.07	85.01±0.64	80.52±0.77	80.64±0.90	94.45±0.21	72.02±1.07	93.39±0.23	82.70±0.63	26.61±0.62
MK-UNet [46]	78.98±1.11	97.48±0.25	82.65±1.02	86.25±1.05	77.87±0.98	94.83±0.24	67.76±0.91	93.77±0.30	80.09±0.50	45.30±2.73
SCAGC-UNet	92.11±0.74	99.09±0.05	94.16±0.63	92.28±0.76	91.98±0.77	97.90±0.16	87.50±1.04	97.32±0.20	92.41±0.60	15.32±1.08

**Table 2:** Performance comparison of different models on the COVID-19 CT dataset.

Method	Rec	SP	Prec	DSC	F2	ACC	IOU_label_	IOU_bg_	MIOU	HD
EMCAD [40]	77.01±0.45	99.76±0.02	80.21±1.25	78.60±0.65	76.36±0.42	99.51±0.05	64.47±0.74	99.49±0.05	82.36±0.39	17.48±0.67
CMUNeXt [41]	69.82±0.15	99.70±0.13	75.45±1.61	69.03±0.17	68.70±0.33	99.28±0.10	56.95±0.17	99.25±0.24	78.10±0.57	30.21±1.14
CCBANet [30]	75.52±0.70	99.71±0.03	77.46±0.75	74.53±0.37	74.61±0.45	99.47±0.05	62.83±0.48	99.44±0.05	81.14±0.26	23.64±0.64
UNet [1]	77.96±0.86	99.76±0.03	81.98±1.09	77.52±1.03	77.34±0.93	99.49±0.06	66.10±1.08	99.47±0.06	82.79±0.57	21.83±1.91
UNeXt [42]	70.73±0.74	99.73±0.03	79.50±0.60	70.98±0.62	70.39±0.69	79.74±0.06	59.34±0.62	99.39±0.07	79.36±0.32	33.24±1.45
ACC-UNet [43]	73.40±2.13	99.41±0.12	76.50±3.34	69.81±2.84	71.35±2.47	99.24±0.14	58.29±3.01	99.20±0.15	78.75±1.58	29.99±3.68
Swin-UNet [44]	68.95±1.16	99.66±0.03	72.92±1.16	67.76±0.78	67.81±0.88	99.26±0.05	54.67±0.77	99.24±0.06	76.96±0.38	34.65±1.02
TransUNet [45]	73.50±1.02	99.78±0.02	82.43±0.97	75.04±0.83	73.72±0.87	99.46±0.05	63.48±0.85	99.44±0.06	81.46±0.45	24.84±1.23
MK-UNet [46]	75.07±0.57	99.75±0.04	80.32±0.90	77.61±0.74	74.58±0.61	99.45±0.07	63.44±0.87	99.43±0.08	81.37±0.47	18.09±0.48
SCAGC-UNet	83.74±0.57	99.76±0.02	82.88±1.21	82.51±0.61	83.06±0.49	99.60±0.91	70.23±0.68	99.58±0.05	85.87±0.37	18.64±1.21

**Table 3:** Performance comparison of different models on the BUSI dataset.

Method	Rec	SP	Prec	DSC	F2	ACC	IOU_label_	IOU_bg_	MIOU	HD
EMCAD [40]	69.18±1.30	97.05±0.16	68.43±1.88	68.80±3.08	66.30±2.35	94.39±0.11	52.44±2.41	93.96±0.15	73.35±1.23	46.26±6.03
CMUNeXt [41]	79.07±0.40	98.00±0.19	80.04±1.11	76.12±1.53	76.99±1.03	96.15±0.09	66.49±1.17	95.69±0.15	81.09±0.61	23.49±3.57
CCBANet [30]	88.12±0.67	97.97±0.26	80.75±3.85	84.30±2.31	85.10±0.96	96.62±0.35	72.86±3.34	96.58±0.39	85.08±1.85	15.16±3.57
UNet [1]	84.61±0.63	98.71±0.25	86.25±2.03	82.98±1.04	83.49±0.85	97.38±0.27	75.28±1.08	97.00±0.26	86.14±0.62	20.99±3.42
UNeXt [42]	78.11±2.80	98.77±0.26	83.47±1.60	77.36±1.74	77.27±2.11	96.66±0.22	67.51±1.41	96.25±0.25	81.88±0.60	31.25±2.34
ACC-UNet [43]	83.70±1.54	97.40±0.04	78.18±1.43	78.07±0.99	80.62±1.10	96.46±0.17	68.39±0.76	95.90±0.19	82.14±0.42	27.51±2.54
Swin-UNet [44]	63.35±8.09	97.34±0.59	68.94±7.18	58.69±10.35	59.97±9.50	94.09±1.23	47.04±12.19	93.55±1.29	70.30±6.74	47.85±20.47
TransUNet [45]	82.13±1.22	98.12±0.05	81.42±0.99	78.79±1.51	79.96±1.34	96.41±0.25	70.50±1.74	95.97±0.24	83.24±0.99	26.66±1.36
MK-UNet [46]	86.73±1.15	97.23±0.46	80.06±2.19	81.24±1.87	83.32±1.63	96.20±0.44	68.40±2.05	95.77±0.43	84.24±1.23	22.65±2.61
SCAGC-UNet	87.07±1.91	98.63±0.16	86.60±1.92	85.70±2.62	86.14±1.70	97.63±1.09	78.10±2.16	97.27±3.53	87.68±1.19	14.87±2.65

**Table 4:** Comparison of computational complexity among different methods.

Method	Flops(G)	Params(M)
EMCAD [40]	1.92	6.53
CMUNeXt [41]	66.59	3.15
CCBANet [30]	7.80	31.57
UNet [1]	589.02	34.53
UNeXt [42]	4.99	1.47
ACC-UNet [43]	532.63	16.77
Swin-UNet [44,45]	102.03	41.37
TransUNet [45]	32.23	105.32
MK-UNet [46]	184.25	315.57
SCAGC-UNet	155.85	16.74

**Table 5:** Component ablation study of graph convolution branches on three datasets.

Method	Rec	SP	Prec	DSC	F2	ACC	IOU_label_	IOU_bg_	MIOU	HD
**Kvasir-SEG**										
RD	92.39±1.91	98.87±0.21	93.80±2.08	92.24±1.87	92.08±1.84	97.88±0.33	87.38±2.06	97.26±0.38	92.28±1.12	15.98±4.11
RDC	92.48±1.69	99.06±0.30	93.88±1.86	92.31±1.74	92.18±1.70	97.87±0.42	87.45±1.89	97.29±0.47	92.37±1.13	14.29±2.56
RDS	92.07±1.80	99.03±0.35	94.04±2.05	92.12±1.96	91.83±1.86	97.80±0.41	87.20±2.17	97.20±0.46	92.20±1.25	15.81±3.07
SCAGC-UNet	92.11±0.74	99.09±0.05	94.16±0.63	92.28±0.76	91.98±0.77	97.90±0.16	87.50±1.04	97.32±0.20	92.41±0.60	15.32±1.08
**COVID-19 CT**										
RD	80.00±3.35	99.28±0.15	58.05±2.41	67.28±3.06	70.40±2.73	99.09±0.17	49.14±2.29	99.06±0.18	74.10±1.23	37.69±4.48
RDC	83.72±1.43	99.58±0.08	67.90±2.75	74.98±2.18	77.08±0.68	99.39±0.08	59.40±1.73	99.37±0.08	79.38±0.90	25.63±2.38
RDS	82.38±1.60	99.57±0.06	68.08±2.10	74.55±2.61	76.00±0.97	99.37±0.07	58.67±1.71	99.35±0.08	79.01±0.89	28.14±2.83
SCAGC-UNet	83.74±0.57	99.76±0.02	82.88±1.21	82.51±0.61	83.06±0.49	99.60±0.91	70.23±0.68	99.58±0.05	85.87±0.37	18.64±1.21
**BUSI**										
RD	84.13±2.31	98.48±1.70	76.06±2.33	79.89±1.97	80.31±2.11	97.08±3.45	66.50±2.21	96.65±4.13	81.57±1.17	17.49±2.06
RDC	86.77±1.69	98.70±0.18	85.93±1.72	86.35±2.06	85.63±1.64	97.14±0.45	76.65±1.87	96.73±0.51	86.69±1.12	15.98±2.11
RDS	89.35±1.91	98.44±2.15	80.83±1.92	85.20±1.76	86.58±1.70	97.60±3.09	72.95±2.16	97.24±3.53	85.05±1.19	17.42±1.97
SCAGC-UNet	87.07±1.91	98.63±0.16	86.60±1.92	85.70±2.62	86.14±1.70	97.63±1.09	78.10±2.16	97.27±3.53	87.68±1.19	14.87±2.65

**Table 6:** Performance comparison of different backbone encoders on three datasets.

Method	Rec	SP	Prec	DSC	F2	ACC	IOU_label_	IOU_bg_	MIOU	HD
**Kvasir-SEG**										
ResR	93.20±0.97	98.59±0.19	92.59±0.86	92.15±0.82	92.47±0.93	97.80±0.28	87.27±1.27	97.20±0.46	92.23±0.75	14.93±3.04
UNetR	92.64±1.25	98.86±0.16	93.28±0.96	92.03±1.12	92.08±1.20	97.76±0.29	87.18±0.14	97.17±0.35	92.17±0.84	15.89±1.13
OURS	92.11±0.74	99.09±0.05	94.16±0.63	92.28±0.76	91.98±0.77	97.90±0.16	87.50±1.04	97.32±0.20	92.41±0.60	15.32±1.08
**COVID-19 CT**										
ResR	81.11±2.28	99.63±0.51	67.12±2.54	73.45±2.43	74.50±2.31	99.46±0.72	58.17±24.78	99.45±0.77	78.81±12.40	26.57±4.84
UNetR	82.59±1.48	99.61±0.04	69.22±1.62	75.32±1.19	76.82±1.09	99.40±0.04	60.34±1.26	99.38±0.05	79.86±0.64	25.00±1.85
OURS	83.74±0.57	99.76±0.02	82.88±1.21	82.51±0.61	83.06±0.49	99.60±0.91	70.23±0.68	99.58±0.05	85.87±0.37	18.64±1.21
**BUSI**										
ResR	87.26±1.89	98.55±1.83	80.29±1.99	83.61±1.93	84.59±1.79	97.27±3.28	72.49±1.94	96.88±3.84	84.68±1.10	18.99±2.09
UNetR	88.88±1.82	98.69±1.85	84.10±1.81	86.42±1.45	86.97±1.74	97.66±3.07	76.83±1.91	97.32±3.49	87.08±1.64	16.86±1.63
OURS	87.07±1.91	98.63±0.16	86.60±1.92	85.70±2.62	86.14±1.70	97.63±1.09	78.10±2.16	97.27±3.53	87.68±1.19	14.87±2.65

## Data Availability

Publicly available datasets were analyzed in this study. These data can be found here: [Kvasir-SEG] (https://datasets.simula.no/kvasir-seg/, accessed on 22 April 2026), [BUSI] (https://www.kaggle.com/aryashah2k/breast-ultrasound-images-dataset, accessed on 22 April 2026), [COVID-19 CT] (https://www.kaggle.com/datasets/mathurinache/mosmeddata-chest-ct-scans-with-covid19, accessed on 22 April 2026).

## Abbreviations

The following abbreviations are used in this manuscript:

ASM	Attention-based Skip Module
BAM	Backward-Aided Module
BUSI	Breast Ultrasound Image Dataset
CCM	Context-Corrected Module
CNN	Convolutional Neural Network
DSC	Dice Score Coefficient
GCN	Graph Convolutional Network
GNN	Graph Neural Network
IOU	Intersection over Union
MIOU	Mean Intersection over Union
HD	Hausdorff Distance
ReLU	Rectified Linear Unit
SC-GCN	Spatial-Channel Graph Convolution
SCAGC-UNet	Spatial-Channel Attention Graph Convolution UNet
SE	Squeeze-and-Excitation
TNT	Transformer-in-Transformer
